# Items

**Published:** 1883-05

**Authors:** 


### Items.

---

The Association of American Medical Editors.—I would respectfully announce that the next annual meeting of the Association of American Medical Editors will be held in the City of Cleveland, Ohio, simultaneously with that of the American Medical Association, on June 5th and 6th, 1883.

Order of exercises. Tuesday, June 5th, 7:15 p. m.—Roll called; reading minutes of previous meeting; president's address and discussion thereon; reports of committees; deferred business; new business; election of members; adjournment.

Wednesday, June 6th, 7:15 p. m.—Roll called; reading minutes of previous meeting; address by Dr. Henry O. Marcy, of Boston; reading of special papers by Dr. John A. Octerlony, of Louisville, Ky., and Dr. Alexander J. Stone, of St. Paul, Minn., discussions of addresses and papers; reports of committees; deferred business; new business; election of officers and members; adjourment.

The subject of the address to be delivered by the President, Dr. N. S. Davis, Chicago, is "The Present Status and Tendencies of the Medical Profession and Medical Journalism." A free discussion upon this important subject is invited, which will be open, not only to members, but to all physicians present. Dr. Marcy's address will be upon the subject of "Journalism devoted to the Protection and Concentration of Medical and Surgical Science in Special Department."

Very respectfully,

J. V. Shoemaker, M.D., Secretary.

---

THE SOUTHERN CULTIVATOR FOR APRIL.—Again has this sterling and popular journal of agriculture paid us its monthly visit, and again are we called on by its excellencies to commend it to the farming people. Time and space are insufficient for a detailed mention of its contents. The most vitality interesting and important of these are "Thoughts for the Month" by Dr. Jones; his Enquiry Department in which answers are given to practical questions; the first letter of a series of letters from the pen of Mr. Dickson; Supreme Court decisions of the several States relating to the interests of agriculture; Letters from the Field; the Patrons of Husbandry; Silk Culture; Fish Culture; Jersey Cattle in the South; Our Sheepfold; Children's Department, and what is a decided novelty with an agricultural journal, a Fashion Department.

The writings of Dr. Jones and Mr. Dickson are worth ten times the price of subscription. These will appear in every number during the year, the former writing as editor—the latter as a contributor. Lives devoted to agriculture, with opportunities for innumerable experiments, prepare these gentlemen to advise Southern farmers and save them from errors they might otherwise commit to their injury. Every farmer should take *The Cultivator* Price per annum, $1.50. Address

JAS. P. HARRISON & CO.,
Atlanta, Ga.

---

A WOMAN in Chicago brought suit against her landlord and his renting agents for damages, as an offset for defects of drainage of a house she occupying and recovered $1000.

In view of this bold act of the enterprising woman of Chicago, we ask for information, whether the law would not be equally protective of the rights of tenants in Atlanta and other cities as well as in Chicago? It may be well for the cause of domestic safety and general sanitation, that Boards of Health make a note of this.

A few years ago it was, a lone woman with her cow and kerosene lamp, of the same great city by the lake, who taught the world a valuable lession in the small beginnings of city conflagrations, and now another woman comes to the front in the same city, with a lesson one thousand dollars strong in sanitation. This is the sort of woman's right which we shall always hold ourself ready to applaud.

---

URINE ANALYSIS.—See, under the proper head, a card from the editor on this important subject.

---

DR. JOHN G. WESTMORELAND.—We direct special attention to the card on another page of this venerable physician. After a long and arduous service in active practice, the Doctor desires to devote himself hereafter to consultations and to an office treatment of certain diseases. We know no physician whose ripe experience and conscientious regard for duty better fit him for such a work.

---

TO THE PROFESSION—ANALYSIS ON URINE.—I am prepared in my office, 64½ Whitehall street, to make qualitative and quantitative analysis of urine and microscopic examination of tissue and fluids, at a moderate compensation. Will be pleased to hear from the profession.

J. H. LOGAN, A.M., M.D.

---

Not being physically able to pursue a general practice of medicine, I hereby decline calls for the treatment of acute diseases, and also chronic affections requiring me to leave my office and exercise much physical labor. Not being financially "able to live without work," I must do an office practice, and desire to confine myself strictly to cases able to call and to diseases with which I have made myself thoroughly familiar, such as chronic stomach, bowel, liver and kidney affections, bronchitis, nasal catarrh and piles.

Office 20 Lloyd street, Atlanta, Ga.

J. G. WESTMORELAND, M.D.

